# The dynamics of the inflammatory response during BBN-induced bladder carcinogenesis in mice

**DOI:** 10.1186/s12967-019-02146-5

**Published:** 2019-11-28

**Authors:** Marina Degoricija, Jelena Korac-Prlic, Katarina Vilovic, Tonci Ivanisevic, Benedikt Haupt, Vinko Palada, Marina Petkovic, Ivana Karaman, Janos Terzic

**Affiliations:** 1 Department of Immunology, School of Medicine, University of Split, 21000 Split, Croatia; 2 Department of Pathology, University Hospital of Split, 21000 Split, Croatia; 3 Department of Physiology and Pharmacology, Karolinska Institutet, 17177 Stockholm, Sweden

**Keywords:** Bladder cancer, Muscle invasive bladder cancer (MIBC), *N*-Butyl-*N*-(4-hydroxybutyl) nitrosamine (BBN), Inflammation, IL18, IFNγ

## Abstract

**Background:**

Bladder cancer (BC) is the most common malignant disease of the urinary tract. Recurrent high grade non muscle invasive BC carries a serious risk for progression and subsequent metastases. The most common preclinical mouse model for bladder cancer relies on administration of *N*-butyl-*N*-(4-hydroxybutyl) nitrosamine (BBN) to mice. BBN-induced tumors in mice recapitulate the histology of human BC and were characterized with an overexpression of markers typical for basal-like cancer subtype in addition to a high mutational burden with frequent mutations in Trp53, similar to human muscle invasive BC.

**Methods:**

Bladder cancer was induced in C57BL/6J male mice by administering the BBN in the drinking water. A thorough histopathological analysis of bladder specimen during and post BBN treatment was performed at 2, 4, 16, 20 and 25 weeks. RNA sequencing and qPCR was performed to assess the levels of expression of immunologically relevant genes at 2 weeks and 20 weeks during and post BBN treatment.

**Results:**

We characterized the dynamics of the inflammatory response in the BBN-induced BC in mice. The treatment with BBN had gradually induced a robust inflammation in the first 2 weeks of administration, however, the inflammatory response was progressively silenced in the following weeks of the treatment, until the progression of the primary carcinoma. Tumors at 20 weeks were characterized with a marked upregulation of IL18 when compared to premalignant inflammatory response at 2 weeks. In accordance with this, we observed an increase in expression of IFNγ-responsive genes coupled to a pronounced lymphocytic infiltrate during the early stages of malignant transformation in bladder. Similar to human basal-like BC, BBN-induced murine tumors displayed an upregulated expression of immunoinhibitory molecules such as CTLA-4, PD-L1, and IDO1 which can lead to cytotoxic resistance and tumor escape.

**Conclusions:**

Despite the recent advances in bladder cancer therapy which include the use of checkpoint inhibitors, the treatment options for patients with locally advanced and metastatic BC remain limited. BBN-induced BC in mice displays an immunological profile which shares similarities with human MIBC thus representing an optimal model for preclinical studies on immunomodulation in management of BC.

## Background

Bladder cancer (BC) is the most common malignant disease of the urinary tract. It is estimated that in 2018 more than half a million people were diagnosed with bladder cancer [1]. Majority of the newly diagnosed BC cases are non-muscle-invasive bladder carcinomas, but high grade, recurrent BC, as well as carcinoma in situ (CIS) rapidly invade the muscle and progress to distant metastases which compel for radical surgical treatment combined with chemotherapeutic approaches in neoadjuvant and adjuvant settings [2]. Muscle invasive bladder cancer (MIBC) is a highly heterogeneous group of tumors which was classified into molecular subtypes that display distinct genomic and transcriptomic profiles similar to those observed in breast cancer [3]. Commonly, MIBCs are characterized as basal or luminal subtypes. Basal tumors, often metastatic at diagnosis, display squamous and sarcomatoid histological attributes, in addition to high EGFR and HIF1 expression, accompanied by epithelial-to-mesenchymal transition cell biomarkers. In contrast, luminal tumors display mostly papillary histologic features and frequently harbor FGFR3, ERBB2, and ERBB3 activating mutations [4]. The Cancer Genome Atlas Network (TCGA) group classified chemotherapy-naïve MIBC into 4 clusters. Clusters I and II correspond to a luminal subtype of BC, whereas Clusters III and IV classify as basal subtypes. Luminal Cluster I tumors, frequently overexpress FGFR3 and display urothelial differentiation markers UPK1A and KRT20, have a better overall survival, whereas Cluster II tumors, which express active P53 gene signature, exhibit resistance to cisplatin. Basal Cluster III, enriched with tumors with mixed squamous morphology, basal markers KRT14, KRT5, and TP63, includes a high cancer stem-content and poor prognosis, while Cluster IV, which shares similarities with claudin-low breast cancer, displays active immunosuppressive phenotype, despite the overall enrichment in the immune gene signature [5].

Immune checkpoint inhibitors targeting the PD-1/PD-L1 axis have been approved for management of locally advanced and metastatic bladder cancer, but low response rates in patients (20–30%) urge for better understanding of mechanisms underlying BC immune escape (reviewed in [6]). Cytotoxic T cell response, characterized with infiltration of CD8+ T cells, chemokines and an interferon γ (IFNγ) expression signature in tumors, are associated with improved survival and better response to checkpoint blockade immunotherapy [7, 8]. T cell inflamed MIBC is characterized with upregulation of immune checkpoint proteins PD-L1, IDO, FOXP3, TIM3, and LAG3, whereas, β-catenin and PPARγ pathways along with activated FGFR3 were described as potential contributors for lymphocyte exclusion in MIBC [9]. Immune transcriptomic analysis of TCGA cohorts revealed a gradient of under-expression of IFNγ associated genes in Cluster I to overexpression in Cluster IV. Basal MIBC Clusters III and IV also displayed increased expression of MHC class II genes which indicate stronger immunogenic nature of basal subtypes of MIBC [10]. In addition to this, recent genomic and transcriptomic analyses of the TCGA bladder cancer cohort, classified bladder cancer into 4 tumor microenvironment immune types (TMITs) based on the PD-L1 expression and the presence of CD8+ cytotoxic lymphocytes (CTLs). Higher overall mutational burden and co-occurrence of mutations in P53 and RB genes were associated with TMIT I group of tumors that present high expression levels of both CD8A and PD-L1 in addition to IFNγ gene signature, unlike TMIT II–IV [11].

The most common approach for investigating bladder cancer in preclinical studies includes the administration of *N*-butyl-*N*-(4-hydroxybutyl) nitrosamine (BBN) to rodents. BBN, a metabolite of the *N*-nitroso-di-*N*-butylamine which is found in tobacco smoke, displays remarkable specificity for bladder carcinogenesis [12]. The carcinogenic effect of BBN relies on alkylating DNA damage and the accumulation of mutations that lead to muscle-invasive bladder cancer (MIBC) in mice [13]. Genomic analysis of BBN-induced cancer in mice revealed a genomic mutational landscape similar to MIBC in human patients. Murine tumors were characterized with a high mutational burden and were driven by mutations in Trp53, as well as expressing the basal subtype cell markers, similar to MIBC in human patients [14].

In this study, we characterized the dynamics of immunological response during BBN induced bladder carcinogenesis in mice by monitoring the leukocyte infiltration burden as well as by expression profiling of immunologically relevant genes during cancer initiation and progression. We observed a robust inflammatory response in the first weeks of BBN treatment that was progressively attenuated until the early progression of CIS, which rapidly progressed to invasive tumors. Moreover, basal-like tumors in mice successfully escaped the immune surveillance thereby presenting an upregulation of immunoinhibitory genes in addition to an upregulated IFNγ gene signature.

## Materials and methods

### Animal work

Experiments were performed on 6 to 8-week-old C57BL/6J male mice. All animal experimental procedures were in accordance with the guidelines provided by the Animal Health Protection Act and the Instructions for Granting Permits for Animal Experimentation for Scientific Purposes and were approved by the Ethics Committee, University of Split, School of Medicine (permit no. 2181-198-03-04-14-0036). Mice were maintained under standard conditions with a 12 h light/dark cycles and provided with free access to tap water and food pellets ad libitum.

### BBN‑induced mouse model of bladder cancer

BBN-treated mice were given tap water containing 0.05% BBN (TCI Europe) for maximum of 12 weeks and afterward replaced with tap water until the end of the experiment. Not treated control group of animals were given tap water throughout the experiment. Mice were sacrificed at 1, 2, 4, 14, 20 and 25 weeks after the beginning of the BBN treatment. One control group included younger mice aged 8 to 10 weeks, age-adjusted to the 2-week timepoint treatment group, whereas the second control group was comprised of mice 26–28 weeks of age, age-adjusted to the 20-weeks treatment timepoint group.

The urinary bladder was removed and cut sagittal into two parts. The part designated for RNA isolation was stored in liquid nitrogen immediately, and the second part was stored in 4% paraformaldehyde for paraffin embedding and histological examination.

### Histological analysis

Paraformaldehyde-fixed tissues were paraffin-embedded and processed for hematoxylin and eosin staining. Two sections from each bladder were evaluated by two expert pathologists in a blinded manner. Histopathological evaluation was performed according to the current World Health Organization classification of human urothelial tumors. The urothelial changes in mice were classified as follows: normal, reactive atypia, dysplasia, carcinoma in situ (CIS), early invasion and invasion. All murine bladder samples were stained with H&E and examined by experienced pathologists in a double-blind manner. Microscopy images were acquired using an Olympus BX43 (Olympus Corporation).

### Immunohistochemistry

Paraffin sections were deparaffinized, hydrated and microwave-heated in either EDTA buffer (pH 8) or citrate buffer (pH 6). The endogenous peroxidase activity was blocked with 3% H_2_O_2_ in methanol and non-specific labeling was blocked by 5% bovine serum albumin. Sections were incubated overnight at 4 °C with the following primary antibodies: anti-CD45 (ab10558, Abcam, 1:500), anti-CD4 (ab183685, Abcam, 1:2000), anti-CD8 (ab203035, Abcam, 1:1000), anti-FoxP3 (ab54501, Abcam, 1:4000), anti-EMBP (sc-33938, Santa Cruz, 1:500), anti-CD163 (ab182422, Abcam, 1:500). For secondary antibodies, HRP-conjugated anti-rabbit (Cat. No. P0448, Dako, diluted 1:500) and anti-goat (sc-2020, Santa Cruz, 1:500) were applied for 1 h at room temperature, followed by standard 3,3′-diaminobenzidine (Cat. No. K3468, Dako) staining procedure. Sections were counterstained with hematoxylin.

### RNA isolation, cDNA library preparation, qPCR array, and next-generation sequencing

RNA was extracted using QIAzol (Qiagen) from bladder tissue according to the manufacturer’s instruction. In the final step, RNA was dissolved in water. One microgram of total RNA was used for cDNA preparation which was performed following the High-Capacity cDNA Reverse Transcription Kit manufacturer’s instructions (Applied Biosystems). Real-time PCR was carried out with Power SYBR Green master mix (Applied Biosystems) using the 7500 Real-Time PCR System (Applied Biosystems). RT2 Profiler Mouse Innate and Adaptive Immune Responses PCR Array (PAMM-052Z) (Qiagen) was used for the analysis of innate and adaptive immune responses during and after BBN treatment. Four mice per study group were tested with RT2 Profiler Mouse PCR Array. Arithmetic mean of Gapdh and Hsp90ab1 was used to normalize gene expression.

The RNA from 9 male mouse bladders at 20 weeks, 8 weeks post-BBN treatment, and 9 control, not treated mice that were matched in sex and age, were pooled into 6 samples (3 RNAs per sample), accordingly. Library was prepared using TruSeq Stranded mRNA LT (Illumina), size distribution of the amplified DNA was assessed with Agilent 2100 Bioanalyzer (Agilent Technologies) and KAPA SYBR^®^ FAST qPCR Kit (KAPA Bio Systems) was used for quantitation of library concentration. Sequencing and FASTQC analysis were performed by Applied Biological Materials Inc. (Richmond, B.C. Canada), single-end 40 million reads were requested and sequenced on Illumina NextSeq500.

### Bioinformatics and statistical analysis

TopHat, Cufflinks, Cuffdiff [15], edgeR [16] and CummeRbund [17] were used to align RNA sequencing reads and analyze differentially expressed genes.

Gene set enrichment analysis (GSEA) was performed using GenePattern (version 3.9.11 prerelease) modules Read_group_trackingToGct (version 0.15) and GSEA (version 19.0.25) on MSigDB gene set collections by performing 1000 gene set permutations [18]. Relative proportions of immune cells from RNA sequencing data were estimated using ImmuCC computational model based on linear support vector regression, on trimmed mean of M values normalized gene counts [19, 20].

GraphPad Prism (version 8.0.2) was used for additional statistical analysis and plotting. Student’s *T* test was used to determine the statistical significance of differentially expressed genes from RT2 PCR Array.

## Results

**Fig. 1:**
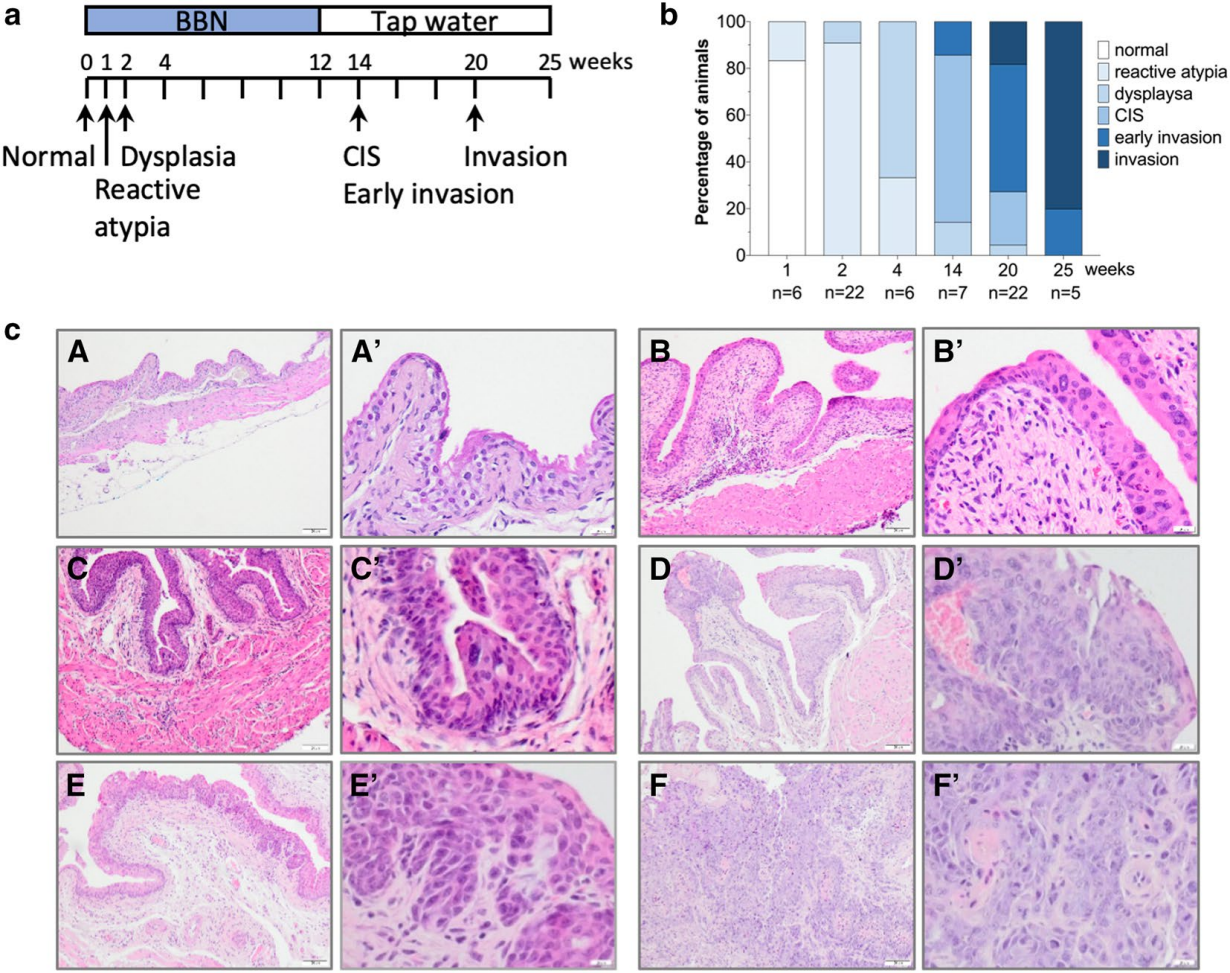
Histopathology of BBN-induced bladder cancer. **a** A schematic representation of BBN treatment chronology. **b** Histologic changes observed throughout different time points of the BBN treatment. **c** Representative images of histologic changes (a, a′) normal urothelial lining, (b, b′) reactive atypia, (c, c′) dysplasia, (d, d′) carcinoma in situ, (e, e′) early invasive tumor, (f, f′) invasive tumor. a–f ×100, a′–f′ ×400, n = number of mice

In order to investigate the dynamics of inflammatory response during BBN-induced bladder carcinogenesis in mice, we performed RNA-seq, RT-qPCR array and a thorough histopathological analysis of bladder specimen at different time points during and post BBN treatment of male C57BL/6 mice. Mice were administered BBN in drinking water for a short period of 1, 2 or 4 weeks and sacrificed in order to determine the initial (acute) inflammatory response in the urinary bladder tissue. To induce tumors in the bladder, mice were treated with BBN for 12 weeks followed by the administration of normal tap water until they were sacrificed at week 14, 20, and 25 (Fig. 1a). One week after BBN treatment only 1 in 6 mice displayed moderate reactive atypia in bladder specimen (Fig. 1b, c B, B′), while all other mice had bladders with normal urothelium (Fig. 1c A, A′). After two weeks of BBN treatment, widespread reactive atypia was present in 20 out of 22 mice, whereas the remaining 2 mice had bladders presenting urothelial dysplasia (Fig. 1c C, C′). After a 4-week BBN treatment, an increased number of mice (4 in 6 mice) had developed dysplasia, while the remaining 2 mice presenting a reactive atypia throughout the urothelial lining. Interestingly, the presence of reactive atypia and dysplasia after a 4-week treatment was focalized, hence the remaining cells were displaying normal cell morphology and tissue architecture (Fig. 1b, c). In addition to this, 4-week BBN treatment followed by additional 16 weeks of tap water, failed to induce tumors at 20 weeks (Additional file 1: Figure S1). The morphologic changes followed the focalized distribution throughout the rest of the treatment timeline, thus, after a 12-week BBN treatment, at week 14, 1 in 7 mice displayed focal urothelial dysplasia, whereas 5 in 7 mice displayed focal CIS (Fig. 1c D, D′) and 1 in 7 mice displayed focal flat lesions with an early invasion in subepithelial connective tissue (Fig. 1c E, E′). Furthermore, at a week 20, around 1 in 22 mice displayed focal urothelial dysplasia, 5 in 22 mice had a focal presence of CIS, 12 in 22 mice had tumors in the stage of an early invasion into subepithelial connective tissue and 4 in 22 mice developed tumors that were invasive (Fig. 1c F, F′). Mice that were sacrificed at a week 25, following a 12-week BBN treatment had all developed invasive tumors with divergent glandular and squamous differentiation, 1 in 5 of these tumors were early invasive, whereas 4 out of 5 tumors were in advanced stages of invasion (Fig. 1b).

**Fig. 2:**
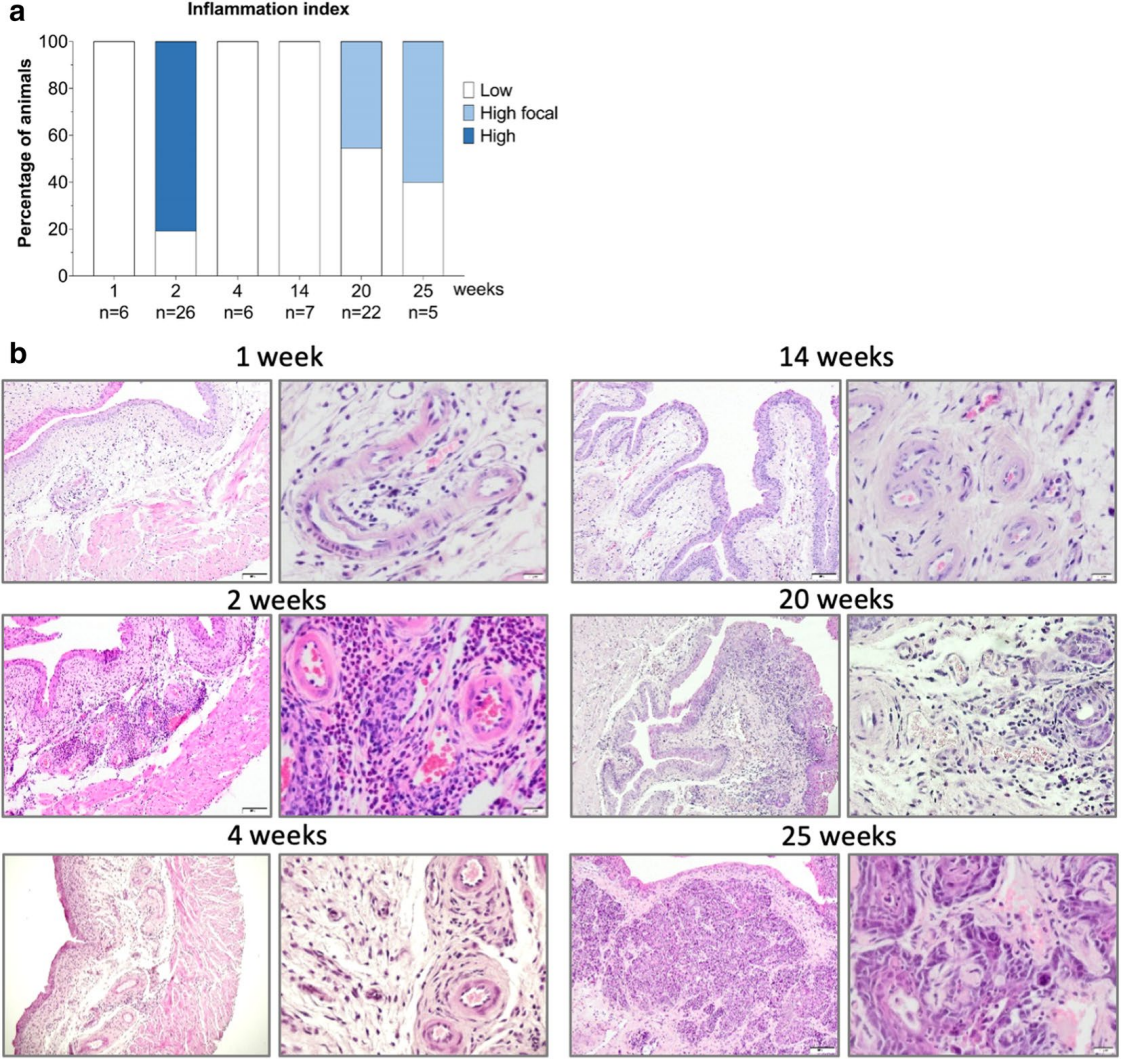
Inflammatory response in BBN-induced bladder cancer. **a** Quantitative assessment of inflammatory burden in mouse bladder specimen during and post BBN treatment. **b** Representative images depicting the extent of the inflammation in different time points of the treatment; (left) cross-section of the bladder, ×100, (right) perivascular space in the subepithelial connective tissue, ×400

After a thorough assessment of the presence of immune cells in all specimens, we determined that after a 1-week BBN treatment, the overall leukocyte burden in the bladder was low and mostly present in the perivascular region of the subepithelial connective tissue, whereas after 2 weeks of treatment, in 21 out of 26 mice there was an evident widespread inflammatory infiltrate throughout the subepithelial connective tissue. Interestingly, after 4 weeks of BBN treatment, there was a striking decrease in bladder inflammatory infiltrate accompanied with increased fibrosis in the subepithelial connective tissue. Similar inflammatory profile was observed at 14 weeks. However, 8 weeks after the end of BBN treatment, at the time point of 20 weeks, 10 in 22 mice displayed pronounced, but focalized inflammatory response in the proximity of the malignant transformation, whereas, at 25 weeks, the presence of focalized inflammation was observed in 3 out of 5 mice (Fig. 2a). Figure 2b displays representative images depicting the extent of an inflammatory response throughout the time points of the BBN treatment.

**Fig. 3:**
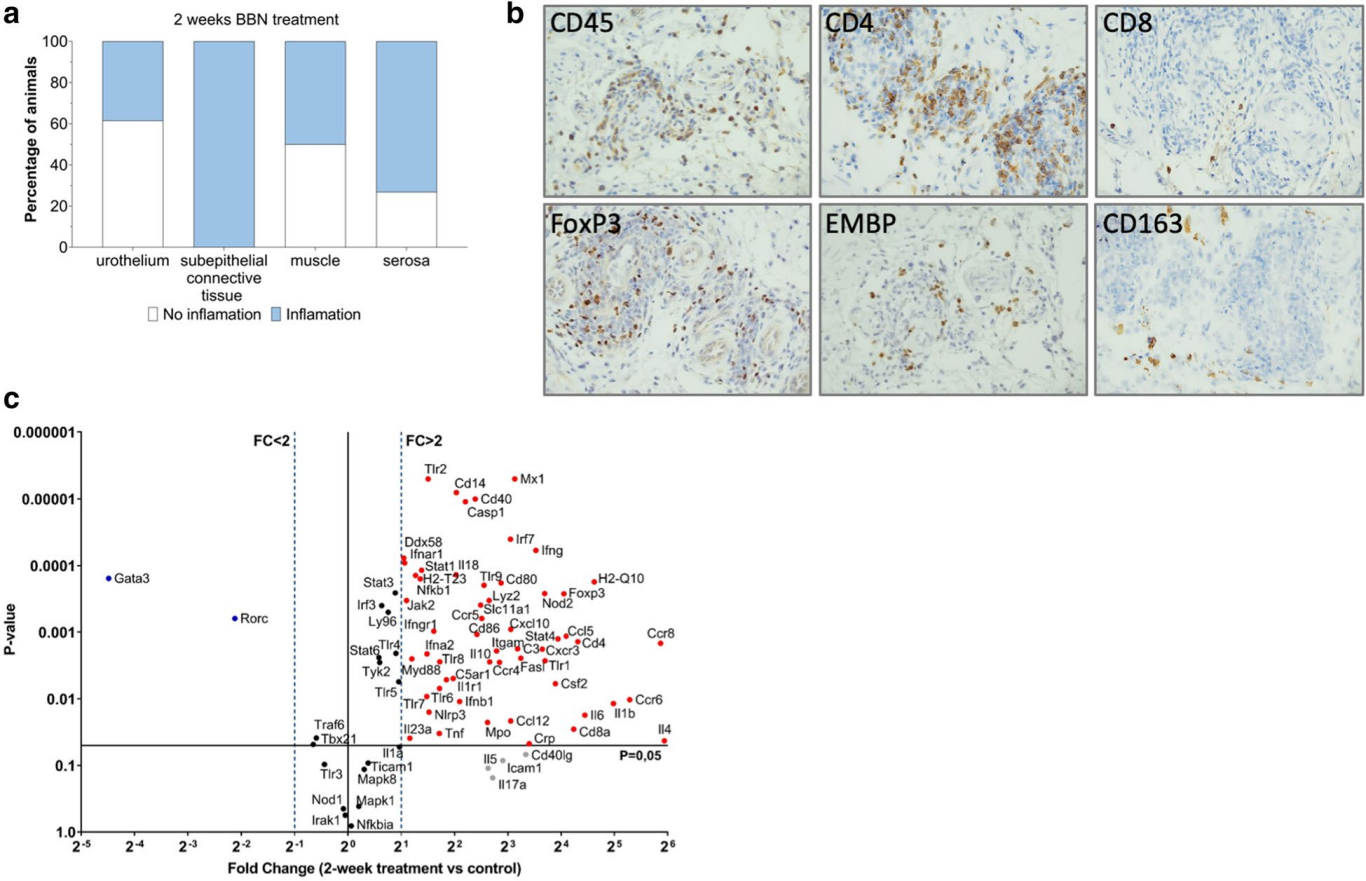
Inflammatory response after 2 weeks of BBN treatment. **a** Quantitative assessment of inflammatory burden in mouse bladder specimen, n = 26. **b** Representative images showing perivascular area in the subepithelial connective tissue stained for leukocytes, different T cell subsets, eosinophils and macrophages. **c** qPCR array analysis of major innate and adaptive immunological response markers, n = 4 per group

Considering the observed differences in qualitative and quantitative attributes of the inflammatory response after 2 weeks of BBN treatment, compared to the inflammatory response at 20 weeks, these time points were chosen for further study. After a 2-week BBN treatment, 10 out of 26 mice had the presence of inflammatory infiltrate in the urothelium, whereas all of the mice had a widespread inflammatory infiltrate throughout the subepithelial connective tissue. The presence of inflammation in the muscle was detected in 13 out of 26 mice, while more than 19 out of 26 mice had detectable immune cells in serosa (Fig. 3a). The inflammatory infiltrate of the subepithelial connective tissue was abundant and widespread with highest concentration of immune cells in perivascular regions of the subepithelial connective tissue. The qualitative and quantitative assessment of inflammatory profile was performed both by immunostaining, as well as by grading the H&E stained slides. We observed a heterogenous leukocyte population, very abundant in granulocytes, macrophages and lymphocytes. Staining for common T cell markers revealed that CD4 + and FoxP3+ lymphocytes were prevalent in comparison to CD8+ lymphocytes (Fig. 3b). The innate and adaptive immune response was further investigated at the level of gene expression. QPCR gene expression profiling revealed a significant upregulation of major proinflammatory cytokines such as Il1∝, Il6, Il18, Il4 as well as IFNγ, TNF∝ and GM-CSF. IL4 was the most prominently expressed cytokine. In addition to this, most of the Toll-like receptors (TLR) tested were upregulated with the exclusion of TLR3. Transcription factors such as NF-κB, Stat3, and FoxP3 were upregulated, whereas, there was an apparent downregulation of Gata3 and Rorc. We observed a very strong upregulation of chemokines Ccl5 and Ccl12 in addition to the upregulated expression of Ccr6, Ccr4 and Ccr8 (Fig. 3c).

**Fig. 4:**
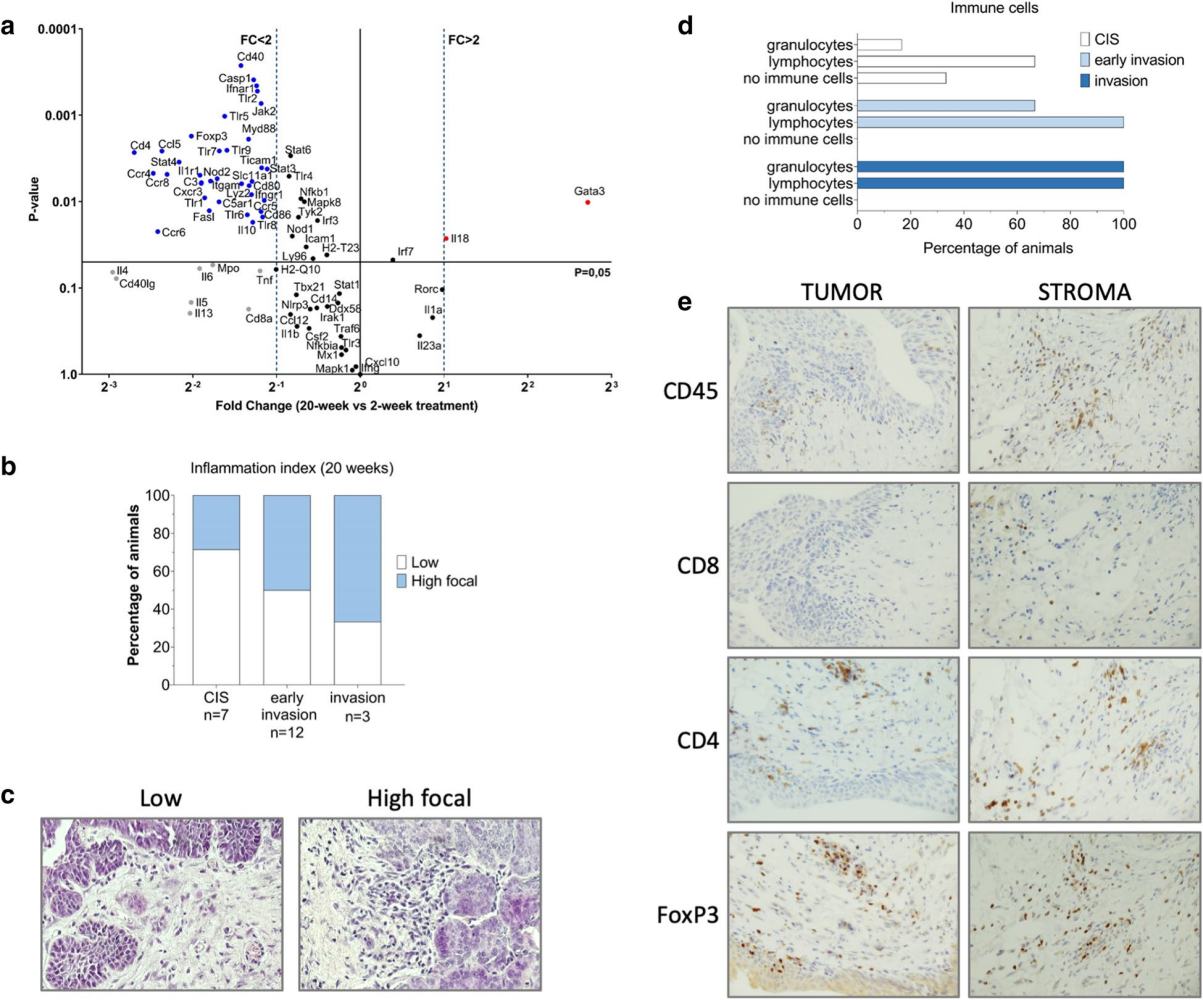
Inflammatory response in tumors at 20 weeks. **a** Differential analysis of expression of the genes involved in innate and adaptive immune response at 2 weeks and 20 weeks, n = 4 per group. **b** Bar plot displaying inflammatory burden during tumor progression. **c** Representative images showing focally high and low inflammatory profile. **d** Differential leukocyte abundance during BC progression. **e** Representative images of T cell infiltrate in tumor and stroma

At week 20, when mice had already developed tumors, we observed a drastic decrease in the extent of inflammation which was also apparent when comparing the expression profile to the profile of 2 weeks of BBN treatment. As shown in Fig. 4a, there was an obvious decline in the level of expression of most inflammation-related genes that were tested, with the exception of Il18 and Gata3. The majority of bladder specimen bearing CIS displayed a low amount of inflammatory infiltrate (5 in 7 specimen), whereas the inflammation was focally more abundant in one half of the specimen with early signs of invasion in subepithelial connective tissue (6 out of 12 specimen). Finally, 2 out of 3 advanced tumors presented high focal inflammation (Fig. 4b, c). The qualitative assessment of the inflammatory profile revealed that, at the early stages of malignant transformation, CIS lesions were infiltrated with very few immune cells. In 4 out of 6 mice few lymphocytes were present at the site of the lesion and in 1 out of 6 mice a small number of granulocytes was detected. However, with the progression of CIS, at the stage of early invasion, all of the mice displayed lymphocytic infiltrate (12 out of 12 mice), whereas granulocytes were present at the site of the lesion in 8 out of 12 animals. The more advanced invasive tumors were inclusive of both lymphocytes and granulocytes (3 out of 3 mice) (Fig. 4d). Immunostaining of early invasive tumors revealed the presence of CD4+, FOXP3+ T and CD8+ cells at the site of the malignant transformation (Fig. 4e).

**Fig. 5:**
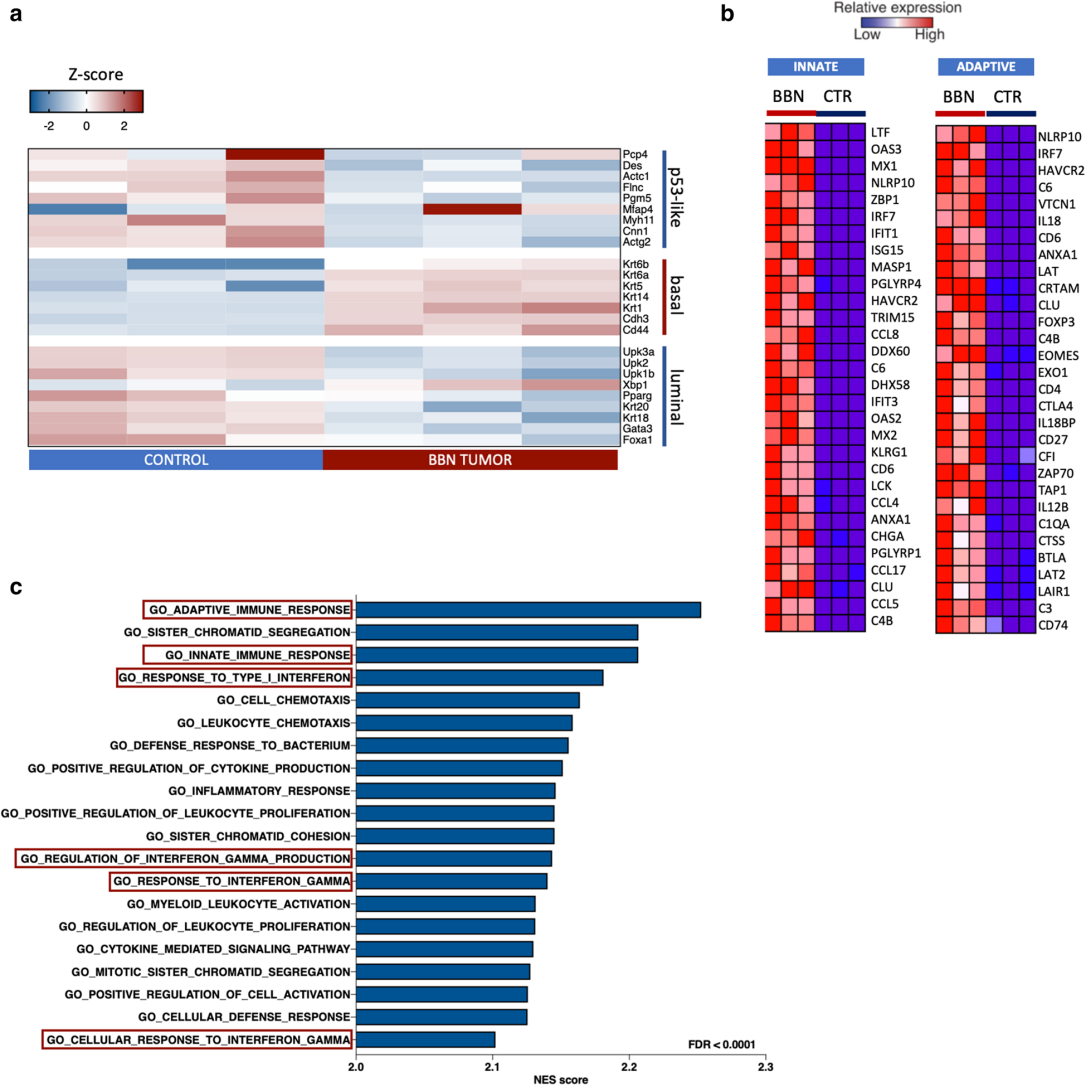
Expression profile of BBN-induced tumors at 20 weeks. **a** Heatmap displaying median based z-scores of gene expression markers for luminal, basal, and p-53-like bladder cancer. **b** Blue-Pink O’gram displaying top 30 differentially expressed genes in tumors relating to innate and adaptive immune response. **c** Results of the GSEA analysis of GO biological processes

**Fig. 6:**
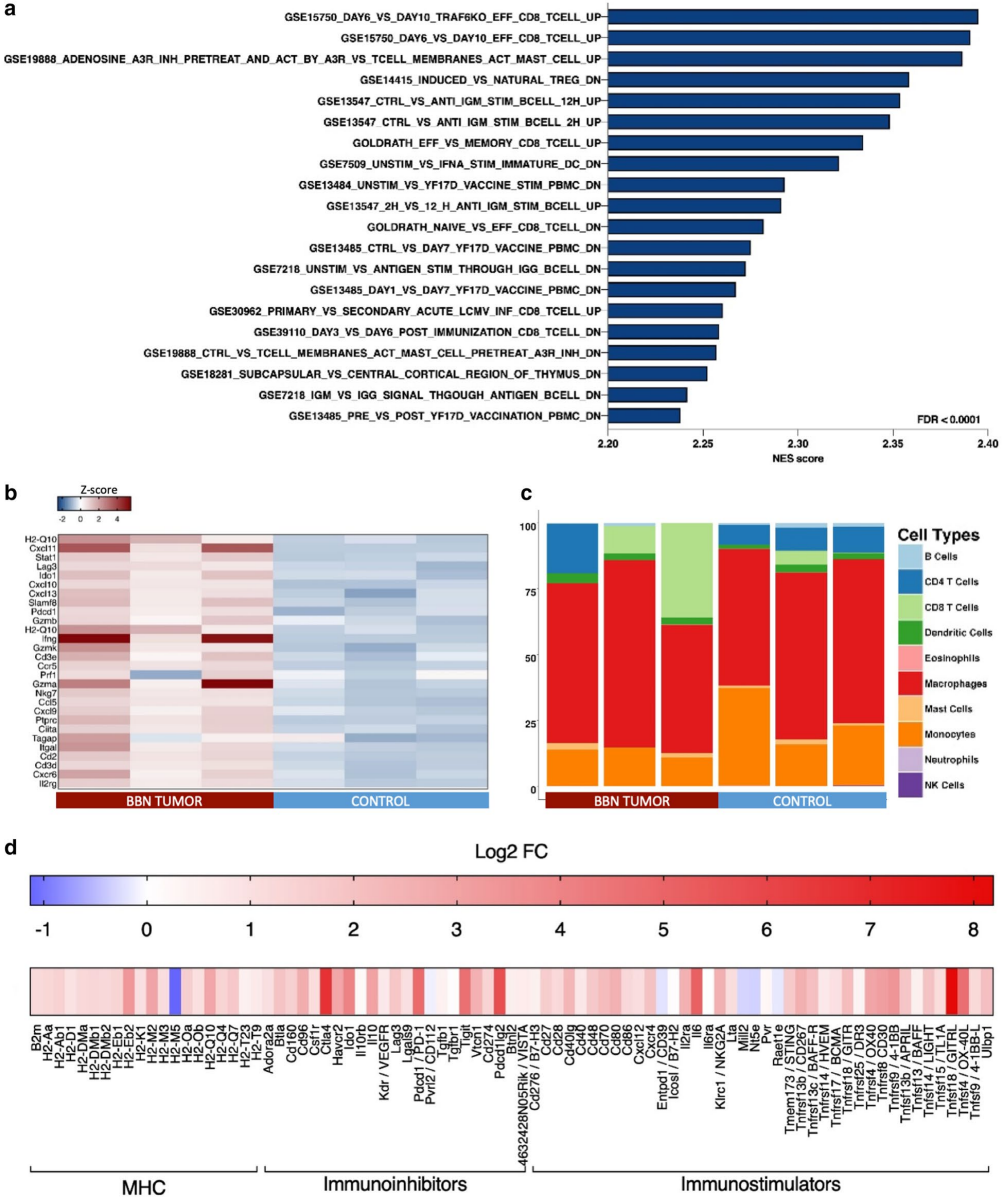
Immunologic profile of BBN-induced tumors at 20 weeks. **a** Immunologic signatures overrepresented in tumors. **b** Heatmap displaying median based z-scores of IFNγ-induced gene expression markers. **c** Seq-ImmuCC prediction of the relative proportions of immune cell populations (y-axis) in tumors and control bladders. **d** Differential expression of MHC and immunomodulatory molecules in tumors

To further investigate expressional profile of BBN induced tumorigenesis, we performed RNA sequencing. We detected 2308 significantly overexpressed genes above 2-fold threshold, 1635 genes were upregulated, and 673 genes were downregulated in the BBN tumor model (FDR < 0.01). Comparison of gene expression in mice treated with BBN for 12 weeks and sacrificed at 20 weeks, to gene expression of non-treated control mice, displayed gene expression profile typical for basal-like keratinizing tumors as previously reported (Additional file 2: Figure S2) [14, 21]. Tumor expression profile included an upregulation of basal cell markers such as Krt5, Krt6, Krt14 and Krt1 as well as Cdh3 and Cd44. The expression of transcription factor Xbp1, typical for luminal MIBC tumors, and the expression of Mfap which is upregulated in P53-like MIBC was variable and upregulated in some of our tumor samples (Fig. 5a). Oncogenic gene signatures enriched in our mouse model showed upregulation of genes associated with deregulation of signaling pathways including polycomb group ring finger proteins (PCGF2), SNF5, RB, P53, mTOR and upregulation of signaling downstream of EGFR receptor among many (Additional file 3: Figure S3). We performed gene ontology enrichment analysis which revealed an overrepresentation of genes involved in both adaptive and innate immune response (Fig. 5b). The leading edge subset of genes involved in innate and adaptive immunity was enriched in genes that are involved in cytokine and MHC class II receptor binding, IL6 receptor signaling, or function as non-membrane spanning protein tyrosine kinases among others (Additional file 4: Table S1). We were interested in gene signatures related to immune response, therefore, GSEA analysis was also performed on Immunological signatures gene set from MSigDB (Fig. 5c). We found an overrepresented number of genes expressed in proliferating CD8+ lymphocytes, genes involved in modulation of mast cells activation, as well as activation of B cells and dendritic cells, pointing out to an ongoing anti-tumor response (Fig. 6a, Additional file 5: Figure S4). Differential expression of genes related to IFNγ response revealed upregulation of genes involved in antigen presentation, chemokine expression, and cytotoxic activity, however, of variable levels among samples (Fig. 6b). Deconvolution method allowed us to estimate the relative proportion of different cell types in mouse bladder samples. Tumors displayed an increase in T cell proportion with a more pronounced CD8+ signature in some of the tumors (Fig. 6c). Granulocytes occurred underrepresented when estimated using RNA sequencing data, however, they were observed during histopathological analysis in majority of invasive tumors (Fig. 4d). We were interested in the expression of MHC, immunostimulatory and immunoinhibitory molecules in BBN tumors so we performed the differential analysis. Both MHC classes of antigens were significantly upregulated and H2-Ebb2, H2-M2 and H2-Q10 had the highest differential expression in tumors. We observed overexpression of important immune checkpoint regulators CTLA-4 and PD-1 as well as the inhibitory ligand PD-L1 which was upregulated 2.5-fold. Immunostimulatory molecules such as IL6, GITRL, OX40 and OX40 ligand, CD30 and NKG2A were among the most differentially expressed (Fig. 6d).

## Discussion

The urinary bladder is lined with pseudo-stratified transitional epithelium (urothelium) which is very quiescent under homeostatic conditions. Upon response to microbial or chemical injury, it undergoes rapid proliferative repair that may be coupled with an inflammatory reaction in the subepithelial connective tissue, muscle, and serosa [22]. In a mouse model of bladder injury with uropathogenic *E. coli*, Mysorekar et al. demonstrate that disruption of urothelial barrier leads to an inflammatory response within 24 h post-infection and that the urothelial regeneration is dependent on the presence of stem and early progenitor cells. In contrast, they observed that intravesicular treatment with protamine sulfate, a chemical that exfoliates luminal barrier cells, does not induce inflammation [23]. Here, we demonstrate that treatment with BBN, a very potent bladder carcinogen, gradually induces a robust inflammation in the first 2 weeks of administration, however, the inflammatory response undergoes silencing in the following weeks of the treatment, until the onset of primary CIS. BBN is a bladder carcinogen that is metabolized mainly in the liver and afterward induces alkylating DNA damage and oxidative stress [12]. Damaged cells are known to release danger-associated molecular patterns as well as pattern-associated molecular patterns which initiate and potentiate noninfectious and infectious inflammatory response which is in line with the expression of proinflammatory cytokines, chemokines and TLRs that we observe already at the 2 weeks of BBN treatment, similar to findings previously described in different cancer models [24]. Gradual downregulation of the inflammatory response during the exposure to BBN might be a consequence of urothelial adaptation via upregulation of metabolic pathways that manage oxidative damage and DNA repair coupled to increased urothelial proliferation [25]. In support of this, we observed an upregulation of IL4, a pro-resolving mediator of inflammation, which was one of the most prominently expressed cytokines at 2 weeks of BBN treatment. IL4 is known for its ability to induce Th2 phenotype of lymphocytes as well as to trigger the switch towards regulatory M2 phenotype of macrophages thereby promoting fibrosis and tissue repair [26, 27]. In line with our findings, a similar contraction of the inflammatory response in the bladder, followed by fibrosis, was also observed in a mouse model of bladder schistosomiasis where upregulation of IL4 was observed 3 weeks post-injection with *S. haematobium* eggs that are well described in the pathogenesis of squamous cell carcinoma of the bladder [28]. The role of IL4 in tumor initiation and progression is not fully understood, however, it was shown that IL4 can promote tumor growth in preclinical models of murine lymphoma [29]. Conversely, IL4 inhibited proliferation of human renal, colon, and breast cancer cells in addition to inducing regression in mouse xenograft models of renal cancer [30, 31]. Furthermore, IL4 coupled with IL13 can promote the proliferation of epithelial cells expressing IL4RA as well as differentiation of adult tissue stem and progenitor cells [32, 33]. IL4RA is expressed on human bladder cancer cell lines and its expression is correlated to tumor stage and grade [34]. High urinary concentrations of IL4 were recently associated with poor recurrence-free survival in patients with bladder cancer, but the exact role of IL4 in bladder regeneration and bladder tumorigenesis still remains elusive [35].

Inflammatory response was recently described in a similar BBN mouse model where acute response via neutrophils was shown to be dependent on FGFR3 signaling in a study by Foth et al. In agreement with them, we observe similar histological changes at 2 weeks, however, at 20 weeks we observe a slightly more advanced tumor stage, which is probably due to a 2-week longer BBN treatment. When compared to an acute 2-week response, they demonstrate a decrease in neutrophil infiltration, after a 10 week-BBN treatment, which is in accordance with an overall decrease in inflammatory response described in our study [36]. Our histological findings are further supported by a decrease in the expression of major inflammatory cytokines and chemokines that are well-described drivers in the setups of acute and chronic inflammation. One interesting finding is the overall upregulation of IL18 in tumors since it was previously shown that serum levels of IL18 were higher in bladder cancer patients than in healthy controls [37]. IL18 is a proinflammatory pleiotropic cytokine and a potent inducer of IFNγ production which in turn activates strong response with CD4+ Th1, CD8+ and NK immune cells [38]. Even though the levels of IFNγ remained unchanged in comparison to the acute inflammatory reaction at 2 weeks, the overall decreased number of focally distributed leukocytes at 20 weeks might suggest the higher local concentration of IFNγ which would account for the early lymphocytic response in early stages of CIS progression into invasive tumors. In several preclinical models, IL18 was found to have antitumor activity, however, its role is possibly tumor-specific [39]. Nevertheless, it is generally accepted that tumors displaying high levels of IL18 and consequently IFNγ, are immunologically more active, thus being potential targets for immune checkpoint-directed therapy [8]. In fact, attempts have been made to improve response to BCG vaccine treatment for non-muscle invasive BC using recombinant IL18-secreting BCG strain which improved the Th1 response in a murine preclinical model in addition to eliciting a better stimulation of human dendritic cells to trigger strong IFNγ secretion by naïve CD4+ lymphocytes [40, 41].

Tumors induced by BBN in WT mice were previously described in lineage-tracing studies to originate from KRT5 positive basal layer cells [42]. The expression profile of BBN tumors in our model was consistent with basal cell markers as well as resembling the basal-like MIBC tumors defined by TCGA study and others [3, 5, 14, 43]. However, we observed a variable, but slightly increased expression of transcription factor Xbp1, an important regulator of MHC class II and ER-induced stress response, in addition to the variable expression of Mfap1 [5, 43]. This finding might be due to the abundance of early invasive tumors in our study group at 20 weeks, when the majority of the urothelial lining still remained well differentiated and non-malignantly transformed. GATA3 is a known breast tumor suppressor and an important marker of urothelial differentiation which is involved in the prevention of bladder cancer progression. We observed the downregulation of GATA3 already after 2 weeks of BBN treatment as well as in tumors, suggesting that dysregulation of GATA3 might be an early event during bladder cancer development in mice and this finding is in line with the high-grade invasive phenotype of BBN-induced tumors [43, 44]. High expression of basal cell markers and loss of luminal cell markers relate BBN-induced tumors in mice with basal-like subtypes, defined by TCGA, of human urothelial carcinoma subtype which are associated with cancer stem-cell expression features. Transcriptomic analysis of immunological profiles of the 4 distinct TCGA MIBC molecular subtypes demonstrated that basal-like clusters III and IV were overexpressing IFNγ associated genes as well as cytotoxic genes. We observed that BBN-induced bladder cancer in mice is characterized with an upregulation of IFNγ responsive genes which could account for the observed lymphocytic infiltrate at the early stages of malignant transformation in bladder. However, similar to human basal-like bladder cancer, murine tumors in our model displayed an upregulated expression of immunoinhibitory molecules such as CTLA-4, PD-L1 and IDO1 which can lead to cytotoxic resistance and tumor escape which was observed at 25 weeks [10]. Comparably, Saito et al. recently demonstrated that BBN-derived (BBN963) subcutaneously-induced syngeneic tumors in immunocompetent mice, closely resemble human bladder cancer. BBN963 tumors were defined with an elevated expression of immune gene signatures but were also characterized with an expression of genes associated to immunosuppression, which made them responsive to a monoclonal antibody against PD-1. In addition to this, transcriptome analysis of MB49, BBN-induced murine bladder cancer cell line, revealed that MB49 cells express highly immunogenic class I and class II peptides which induced strong IFNγ response in T cells stimulated by neoantigen peptide-pulsed dendritic cells [45]. It is known that IFNγ itself promotes expression of PD-L1 and PD-L2 in tumor cells, as well as in stromal cells, including immune infiltrating cells, thereby suppressing the effector functions of tumor-specific T cells and NK cells via PD-1 [7, 46]. In addition to this, IFNγ was recently described as an upregulator of CTLA-4 in melanoma cells, another long-established immune checkpoint inhibitor [47]. Finally, we observed upregulation of expression of the immunoregulatory tryptophan-metabolizing enzyme IDO1, an inducer of regulatory T cells which have potent immunosuppressive abilities [48]. IFNγ treatment was demonstrated to some extent beneficial when intravesically instilled in patients with superficial bladder cancer, but its role in the progression of CIS and basal-like bladder carcinoma is not described well [49]. Immune gene expression profiling is becoming very useful in determining biomarkers for successful immune checkpoint inhibition. Expression of IFNγ inducible molecular signature was associated with good overall response to atezolizumab in bladder cancer patients [50]. Finally, a study by Ayers et al. defined an expression of 18-gene set as a useful for evaluation of IFNγ signature that could predict the outcome of PD-1 blockade [8]. Here we observe that BBN-induced BC in mice is characterized with an upregulated expression of IFNγ inducible genes thus presenting an optimal model for studies of immune escape during bladder cancer initiation and progression.

## Conclusions

Despite the recent advances in bladder cancer therapy which include the use of checkpoint inhibitors, the treatment options for patients with locally advanced and metastatic BC remain limited. We have characterized the dynamics of immunological response during BBN induced bladder carcinogenesis in mice by monitoring the leukocyte infiltration burden as well as by expression profiling of immunologically relevant genes during cancer initiation and progression. BBN-induced BC in mice displays an immunological profile which shares similarities with human MIBC and is characterized with an upregulated expression of IFNγ inducible genes thus representing an optimal model for preclinical studies on immunomodulation in management of BC.

## Supplementary information

**Additional file 1: Figure S1.** Representative images of Ki67 and H&E stained mouse bladder after 4 weeks of BBN treatment and followed by 16 weeks of tap water.

**Additional file 2: Figure S2.** Heatmap displaying median based z-scores of typical gene expression markers for basal keratinizing-like tumors in mice.

**Additional file 3: Figure S3.** Results of GSEA analysis on oncogenic signatures gene set.

**Additional file 4. Table S1.** Analysis of the leading edge subset of genes involved in innate and adaptive immunity.

**Additional file 5: Figure S4.** Snapshot of top 16 GSEA analysis results on immunological signatures gene set.

## Data Availability

Additional file 1: Figure S1, Additional file 2: Figure S2, Additional file 3: Figure S3, Additional file 5: Figure S4 can be found at http://. All raw data supporting our findings is available on request. Raw sequencing data was submitted to NCBI SRA Database as Bioproject: PRJNA587619.

## Abbreviations

BBN: *N*-butyl-*N*-(4-hydroxybutyl) nitrosamine
BC: bladder cancer
BSA: bovine serum albumin
CIS: carcinoma in situ
CTLs: cytotoxic lymphocytes
FDR: false discovery rate
GSEA: gene set enrichment analysis
IFNγ: interferon γ
MHC: major histocompatibility complex
MIBC: muscle invasive bladder cancer
TCGA: The Cancer Genome Atlas
TLR: Toll-like receptor
TMIT: tumor microenvironment immune type
WHO: World Health Organization
