# Supplementary figures and images for: The dynamics of the inflammatory response during BBN-induced bladder carcinogenesis in mice
Source: J Transl Med. 2019 Nov 28;17:394. doi: 10.1186/s12967-019-02146-5 (PMC6883615; doi:10.1186/s12967-019-02146-5)

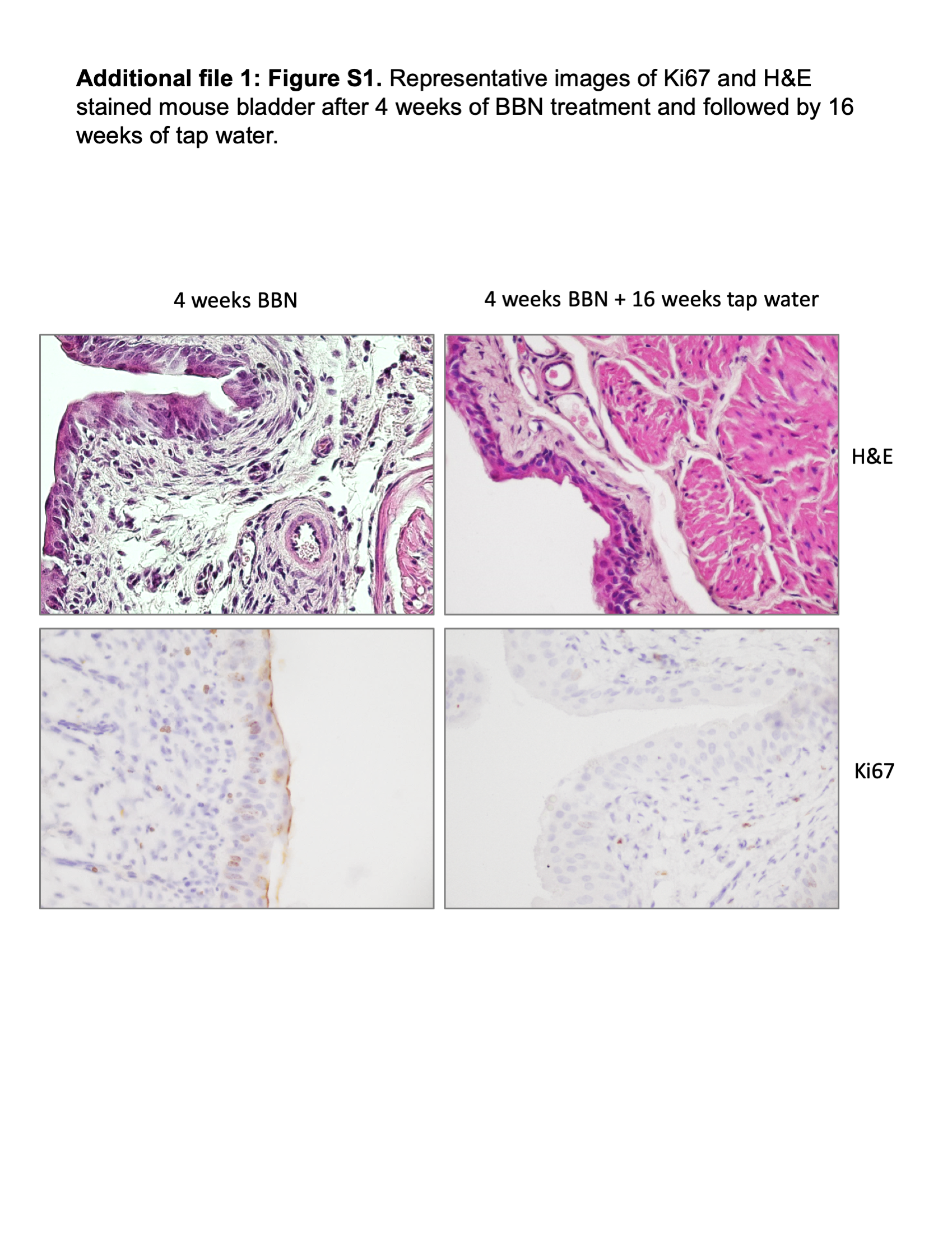

Supplement: Supplementary file 1 — Additional file 1: Figure S1. Representative images of Ki67 and H&E stained mouse bladder after 4 weeks of BBN treatment and followed by 16 weeks of tap water. [file 12967_2019_2146_MOESM1_ESM.tiff]

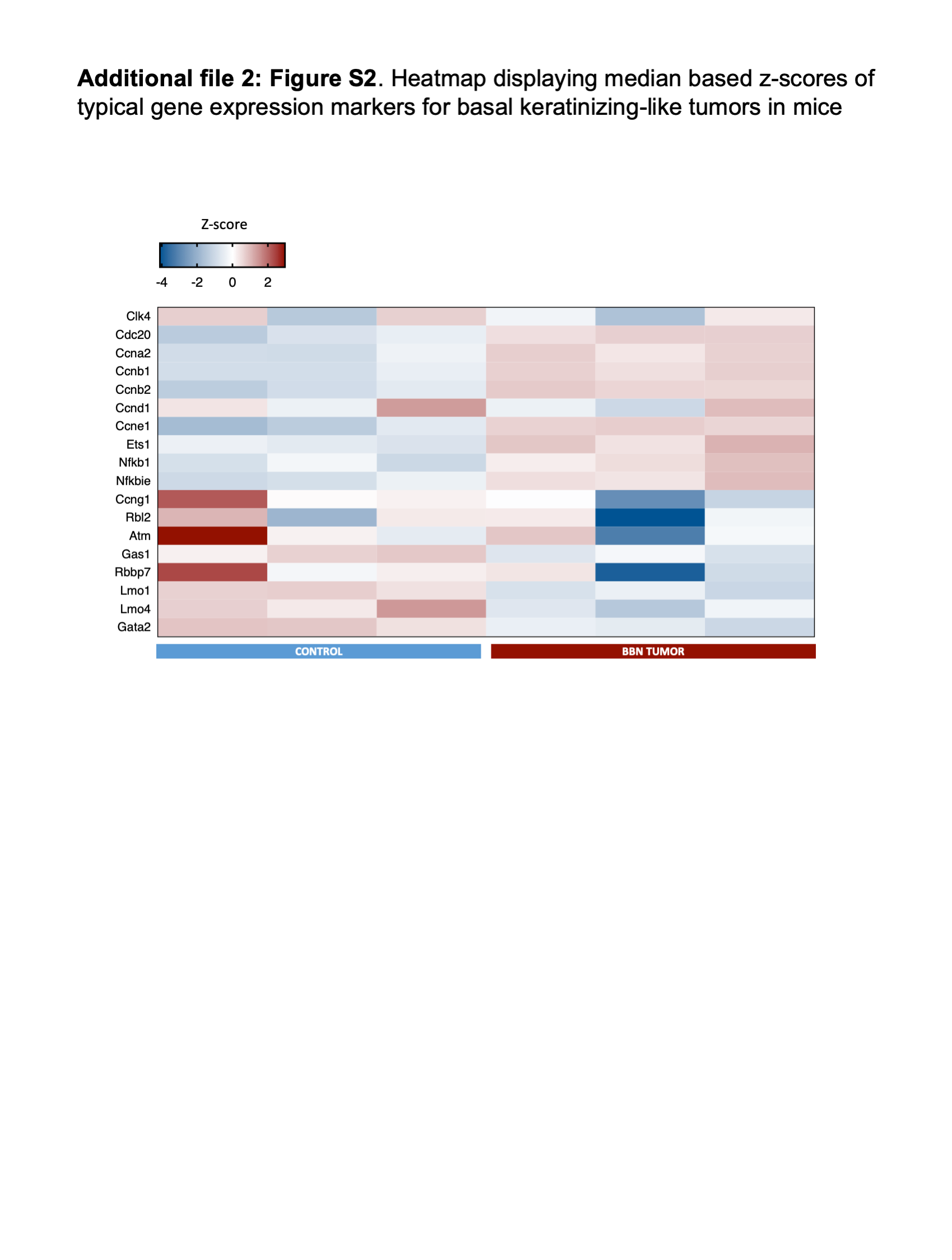

Supplement: Supplementary file 2 — Additional file 2: Figure S2. Heatmap displaying median based z-scores of typical gene expression markers for basal keratinizing-like tumors in mice. [file 12967_2019_2146_MOESM2_ESM.tiff]

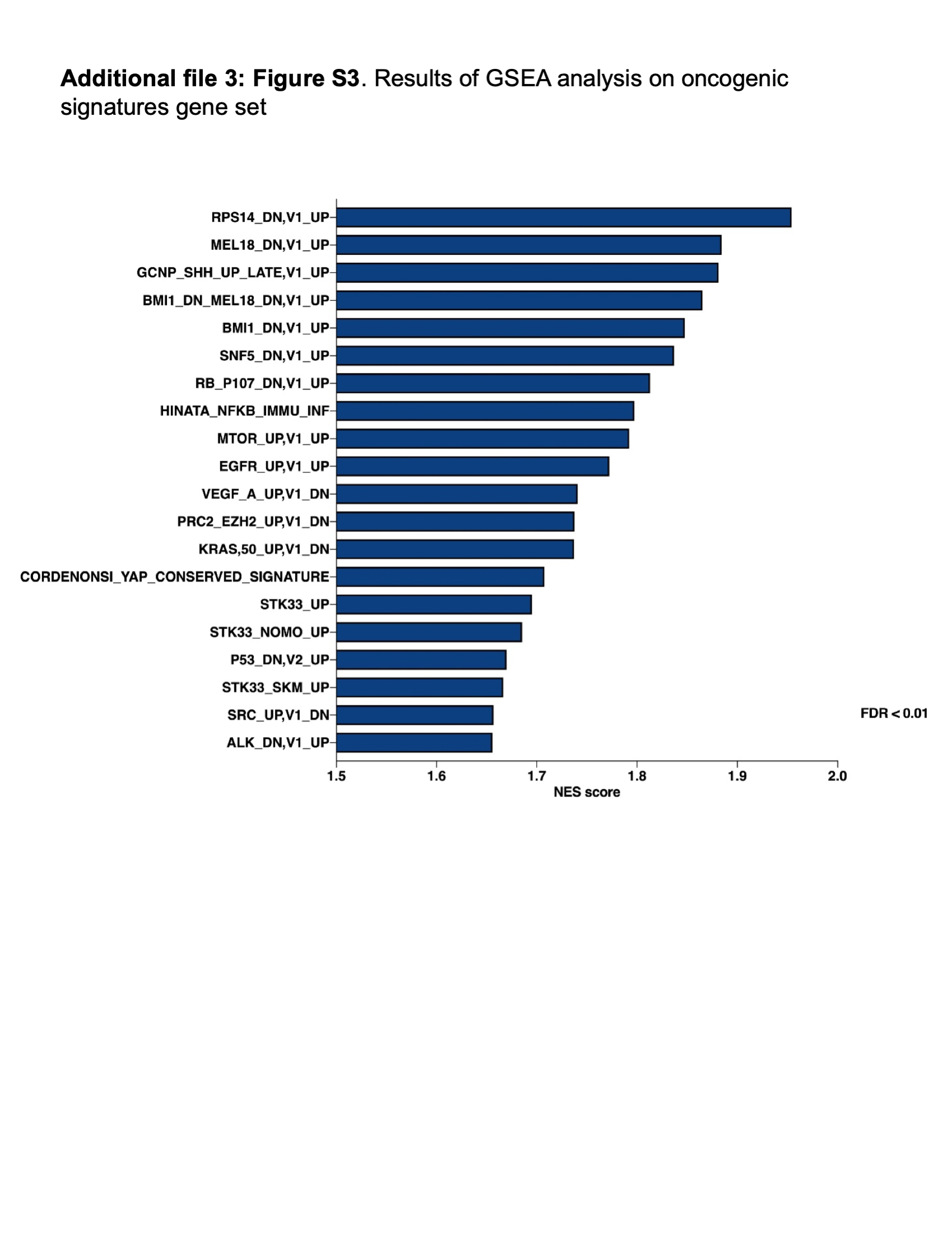

Supplement: Supplementary file 3 — Additional file 3: Figure S3. Results of GSEA analysis on oncogenic signatures gene set. [file 12967_2019_2146_MOESM3_ESM.tiff]

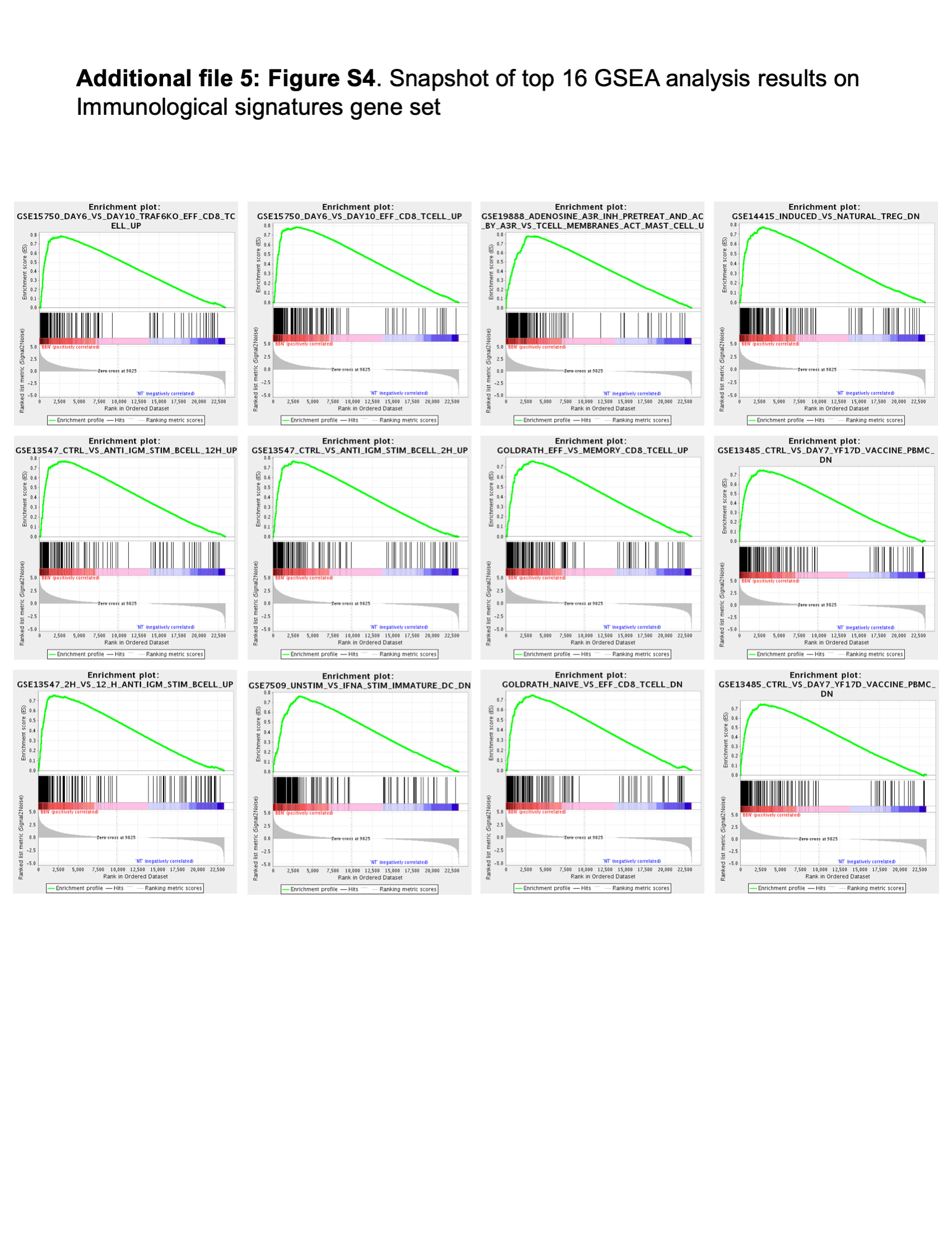

Supplement: Supplementary file 5 — Additional file 5: Figure S4. Snapshot of top 16 GSEA analysis results on immunological signatures gene set. [file 12967_2019_2146_MOESM5_ESM.tiff]
